# Participation in physical activity in patients 1–4 years post total joint replacement in the Dominican Republic

**DOI:** 10.1186/1471-2474-15-207

**Published:** 2014-06-16

**Authors:** Scott A Elman, Yan Dong, Derek S Stenquist, Roya Ghazinouri, Luis Alcantara, Jamie E Collins, Carolyn Beagan, Thomas S Thornhill, Jeffrey N Katz

**Affiliations:** 1 Orthopedic and Arthritis Center for Outcomes Research, Brigham and Women’s Hospital, 75 Francis St, Boston, MA 02115, USA; 2 Department of Orthopedic Surgery, Brigham and Women’s Hospital, 75 Francis St, Boston, MA 02115, USA; 3 Harvard Medical School, 25 Shattuck St, Boston, MA 02115, USA; 4 Department of Biostatistics, Boston University School of Public Health, 801 Massachusetts Avenue, Boston, MA 02118, USA; 5 Department of Rehabilitation Services, Physical Therapy, Brigham and Women’s Hospital, 75 Francis Street, Boston, MA 02115, USA; 6 Department of Orthopedic Surgery, Hospital General de la Plaza de la Salud, Santo Domingo, Dominican Republic; 7 Department of Epidemiology, Harvard School of Public Health, 677 Huntington Ave, Boston, MA 02115, USA; 8 Division of Rheumatology, Immunology, and Allergy, Brigham and Women’s Hospital, 75 Francis Street, Boston, MA 02115, USA

**Keywords:** Knee arthroplasty, Hip arthroplasty, Osteoarthritis, Rheumatoid arthritis, Global health, Dominican Republic

## Abstract

**Background:**

To address both the growing burden of joint disease and the gaps in medical access in developing nations, medical relief organizations have begun to launch programs to perform total joint replacement (TJR) on resident populations in developing countries. One outcome of TJR of particular interest is physical activity (PA) since it is strongly linked to general health. This study evaluates the amount of postoperative participation in PA in low-income patients who received total joint replacement in the Dominican Republic and identifies preoperative predictors of postoperative PA level.

**Methods:**

We used the Yale Physical Activity Survey (YPAS) to assess participation in postoperative PA 1–4 years following total knee or hip replacement. We compared the amount of aerobic PA reported by postoperative TJR patients with the levels of PA recommended by the CDC and WHO. We also analyzed preoperative determinants of postoperative participation in aerobic PA in bivariate and multivariate analyses.

**Results:**

64 patients out of 170 eligible subjects (52/128 TKR and 14/42 THR) who received TJR between 2009–2012 returned for an annual follow-up visit in 2013, with a mean treatment-to-follow-up time of 2.1 years. 43.3% of respondents met CDC/WHO criteria for sufficient participation in aerobic PA. Multivariate analyses including data from 56 individuals identified that patients who were both younger than 65 and at least two years postoperative had an adjusted mean activity dimensions summary index (ADSI) 22.9 points higher than patients who were 65 or older and one year postoperative. Patients who lived with friends or family had adjusted mean ADSI 17.2 points higher than patients living alone. Patients who had the most optimistic preoperative expectations of outcome had adjusted mean ADSI scores that were 19.8 points higher than those who were less optimistic.

**Conclusion:**

The TJR patients in the Dominican cohort participate in less PA than recommended by the CDC/WHO. Additionally, several associations were identified that potentially affect PA in this population; specifically, participants who are older than 65, recently postoperative, less optimistic about postoperative outcomes and who live alone participate in less PA.

## Background

Arthritis is a common cause of pain and functional limitation that affects hundreds of millions of people worldwide [1]. The burden of joint disease is growing in developing nations, whose populations are living longer because of declining mortality due to infectious diseases and other causes of early mortality [2,3]. As there are no validated disease-modifying medications for the treatment of osteoarthritis (OA), such as the disease-modifying medications used for the treatment of rheumatoid arthritis (RA), total joint replacement (TJR) is the treatment of choice for OA patients with severe pain, functional limitation and structural damage for whom conservative treatment has not helped [3-8].

Total joint replacement is a common and cost-effective operation in the US [8]. In developing countries, however, access to TJR is limited due to high cost and lack of available resources and appropriately trained surgeons. To address both the growing burden of joint disease in developing nations and gaps in access to treatment, medical relief programs have been launched in developing countries to perform TJR on resident populations and to transfer critical knowledge and skills to personnel at host facilities [9].

One such program, Operation Walk (Op Walk) Boston, has been providing TJRs to patients in the Dominican Republic since 2008. Op Walk Boston established a research program to evaluate the outcomes of TJR performed by the Op Walk Boston team in a Dominican sample with advanced arthritis [10].

One TJR outcome of particular interest is physical activity (PA). TJR is capable of providing patients with dramatic increases in functional capacity, but it is unclear whether this increase in capacity translates to transformative increases in PA [11,12]. Although the ability of TJR to increase functional capacity in Op Walk Boston’s Dominican population is well documented [10], the extent that Op Walk patients engage in PA postoperatively has not been assessed. This is of interest because PA has been shown to translate into general health gains including reduced burden of cardiovascular disorders, Type 2 diabetes, and certain cancers [13-18]. Additionally, physical inactivity accounts for 6% of deaths globally [19]. Furthermore, regular PA has been shown to reduce fracture risk and improve strength, flexibility and balance in older adults [20-23].

The importance of regular PA has long been recognized. In 2008, the Centers for Disease Control (CDC) released its *Physical Activity Guidelines for Americans*, which established targets for total amounts of PA as either moderate-intensity aerobic PA for at least 150 minutes per week or vigorous-intensity aerobic activity for at least 75 minutes per week in bouts of at least 10 minutes. In 2010, the World Health Organization (WHO) adopted the CDC’s 2008 guidelines as its *Global Recommendations on Physical Activity for Health*, indicating that these PA recommendations were reasonable for global adult populations [24-27].

In this report, we evaluate the PA level of low-income patients from the DR who received TJR during Op Walk Boston’s 2009–2012 trips; we benchmarked this against the levels of PA recommended by the CDC and WHO; and we analyzed potential preoperative determinants of postoperative participation in PA.

## Methods

### Setting

The Dominican Republic occupies the eastern two-thirds of the island Hispaniola. Roughly 34% of the Dominican Republic’s 10.2 million citizens currently live below the international poverty line [28]. All citizens are covered by a public healthcare system; since only basic healthcare needs are included, advanced treatments such as TJR are generally not accessible to the poor. To address the needs of those without access to services beyond basic healthcare, the Op Walk Boston team travels annually to the Hospital General de la Plaza de la Salud (HGPS) in Santo Domingo, Dominican Republic to perform TJR for qualifying patients.

### Study population

Each year since 2008, orthopedic surgeons at HGPS have assembled a group of low-income patients with advanced, symptomatic arthritis. Patients present to the Orthopedic Department of HGPS in the summer and autumn months preceding the spring mission. The orthopedic team at HGPS performs a clinical evaluation including physical exam, history, and plain radiographs. If further consultation is needed (e.g. by a cardiologist) the orthopedist requests the consultation. In the late fall, the HGPS and Boston teams meet to make the final decisions about surgical eligibility. When the Boston team arrives, the patients are evaluated one final time in a preoperative clinic and then patients are scheduled to receive surgery during the week the team is on site. Patients are operated upon by an attending surgeon from the Op Walk Boston team, sometimes with a colleague from HGPS. Bilateral procedures are performed in the same anesthesia. Length of stay is generally three to four days. Patients see a physical therapist three times a week for the first two postoperative weeks. Beyond two weeks postoperatively, there is no scheduled contact with the surgical or rehabilitative teams unless problems arise. Patients are encouraged to continue their PT regimens at home.

### Intervention

All patients in the cohort discussed here underwent unilateral or bilateral hip or knee arthroplasty in 2009, 2010, 2011 or 2012. The patients were re-evaluated in a follow-up clinic in 2013, one to four years postoperatively. The follow-up survey instrument was administered to patients in the follow-up clinic. Volunteers were available to assist those who had difficulty with reading and filling out the forms.

### Data collection procedures

During the preoperative evaluation, research associates invited patients to complete preoperative questionnaires assessing pain, function, demographics and other domains. These patients are invited to return for annual follow-up appointments during the week that the aid team is in residence.

### Survey instrument

#### Preoperative assessment

Each patient enrolled in the program between 2009–2012 was asked to complete a baseline survey in Spanish that consisted of several reliable and validated measures of health related quality of life, as well as items on demographic features and patient expectations of surgery [29]. Specifically, we used the Pain and Functional Status Scales of the Western Ontario and MacMaster Universities Arthritis Index (WOMAC), re-scaled from 0–100 with 100 being the worst, and the Physical Activity Scale of the Short Form (36-item) Health Survey (SF-36). Patients who underwent bilateral surgery were not asked about each joint individually. Each of these scales has been shown to be reliable, particularly in elderly populations and in persons with OA [30,31]. Crohnbach’s alpha coefficients were previously reported to equal or exceed 0.75 in the Dominican cohort for the WOMAC and SF-36 subscales included in our surveys [10,32]. Regarding expectations, we asked patients to indicate the likelihood of pain relief following surgery and the likelihood of a serious complication. Each of these items had five ordinal responses.

#### Postoperative assessment

During Op Walk Boston’s 2013 mission, patients who received TJR from 2009–2012 were invited to return to the follow-up clinic. These patients were asked to complete a follow-up survey that included the same scales as the preoperative survey. In addition, in 2013, the Yale Physical Activity Survey (YPAS) was added to the battery of instruments administered to participants [33]. The YPAS is a reliable tool that measures household, exercise, and recreational components of PA in older adults and has been validated in Spanish-speaking populations [34]. The YPAS contains two parts. Part I asks respondents to indicate how many hours per week they spend participating in a variety of activities – two indices can be calculated from this; a total time summary index (TTSI) and an energy expenditure summary index (EESI). The latter takes into account the various intensities of the checklist of activities. Part II asks respondents to indicate participation in five specific PA categories in the last month – vigorous activity, leisurely walking, moving, standing, and sitting – each of which is then multiplied by a weighting factor corresponding to intensity; the final result is an activity dimensions summary index (ADSI) [35].

The surveys administered to the Dominican patients were written in Spanish. We used previously published Spanish versions of the SF-36 and WOMAC indices [36,37]. All other items were translated by bilingual researchers of this study. Our translation of the YPAS was done according to WHO standards [38,39].

### Preoperative clinical characteristics

We calculated preoperative scores for WOMAC pain and function status scales and SF-36 physical activity and mental health status scales. We also characterized each patient’s lower extremity joint burden by summing the number of joints (left hip, right hip, left knee, and right knee) the respondent indicated as being at least “moderately” painful. We created an additive “optimism” variable that combined patients’ expectations of the probability of pain reduction and the probability of complication after operation. Each of these variables was skewed toward highly optimistic responses (Table 1). To create a readily interpretable variable, patients were considered “most optimistic” if they believed that they had a >90% probability of pain relief and a <1% chance of a severe surgical complication.

**Table 1:** Demographic data of Op Walk Boston patients from 2009–2012 who returned for follow-up and completed the YPAS in 2013

**Preoperative demographic characteristics**		
	**Mean (SD)**	**Range**
Age (n: 64)	61.1 (13.9)	21-80
	**Number**	**Percent**
Gender (n: 64)		
Female	51	79.7
Male	13	20.3
**Socioeconomic characteristics**		
Marital status (n: 62)		
Single	19	30.7
Married	22	35.5
Divorced, separated, or widowed	21	33.9
Living situation (n: 62)		
Alone	6	9.7
With family or friends	56	90.3
Work status (n: 63)		
Working	10	15.9
Not working	53	84.1
Education (n: 62)		
Less than secondary school	25	40.3
Secondary school, but did not graduate	25	40.3
Graduated from secondary school or beyond	12	19.4
**Surgery information**		
Procedure (n: 64)		
Unilateral	38	59.4
Bilateral	26	40.6
Joint (n: 66)*		
Knee	52	81.3
Hip	14	21.9
Year (n: 64)		
2009	8	12.5
2010	13	20.3
2011	22	34.4
2012	21	32.8
**BMI (n: 55)**		
<30	42	76.4
30-35	9	16.4
≥35	4	7.3
**Self-rated health (n: 61)**		
Excellent or very good	17	27.9
Good	20	32.8
Fair or Poor	24	39.3
**Perception of probability of surgical complication (n: 61)**		
<1% chance of major complication	43	70.5
1-5% chance of major complication	8	13.1
5-10% chance of major complication	9	14.8
10-20% chance of major complication	1	1.6
More than 20% chance of major complication	0	0
**Perception of probablity that surgery will relieve pain (n: 63)**		
>90% chance of pain relief	48	76.2
70-90% chance of pain relief	11	17.5
50-70% chance of pain relief	4	6.4
25-50% chance of pain relief	0	0
<25% chance of pain relief	0	0
**Preoperative scores (n: 64)**	**Mean (SD)**	**Range**
WOMAC pain**	59.9 (23.1)	10-100
WOMAC function**	62.0 (22.0)	8.8-100
SF-36 mental health***	78.0 (19.3)	12-100
SF-36 physical function***	18.6 (19.4)	0 - 85
**Postoperative scores (n: 64)**		
WOMAC pain**	20.2 (23.6)	0-90
WOMAC function**	16.6 (16.3)	0-62
SF-36 physical function***	68.4 (24.7)	10-100

*Percentages do not add up to 100% because two patients had both TKR and THR.

**possible range 0–100, 100 = worst.

***possible range 0–100, 100 = best.

### Study outcomes

We examine two related outcomes. First we calculated postoperative YPAS physical activity scores for patients. Part I scores were reported as both total time summary index (TTSI) and energy expenditure summary index (EESI). Part II scores were calculated and summarized in an activity dimensions summary index (ADSI). Second, from the calculated YPAS part II scores, we created a binary indicator reflecting whether or not subjects met the CDC/WHO recommendations for participation in aerobic PA.

### Statistical methods

#### Statistical tests

We used ANOVA or Fisher’s Exact Test as appropriate to assess the bivariate associations between PA level (both as a continuous outcome measured by the ADSI and as a binary outcome – meets/does not meet CDC criteria), and several covariates obtained preoperatively. These included age, year of surgery, sex, BMI, smoking status, alcohol consumption, marital status, living situation, work status, preoperative expectations of surgery, and WOMAC and SF-36 scores.

The distribution of ADSI data points was examined with a histogram and Q-Q plot and did not substantially deviate from normal, supporting use of linear regression. We constructed a multivariate linear model to identify independent associations between baseline characteristics and postoperative level of participation in aerobic PA. We advanced to the multivariable model those covariates that had an odds ratio of ≥1.5 or ≤ 0.67 in association with the binary outcome (meets CDC criteria) or that were statistically significantly associated with the continuous ASDI variable. Due to sample size limitations, we used the continuous ADSI score as the dependent variable for the multivariate model rather than the binary indicator (meets/does not meet CDC criteria). We employed a backward selection process with a threshold p value of 0.3. This value was chosen because we did not wish to exclude variables with meaningful measures of effect as demonstrated by the odds ratio simply on the basis of the bivariate p-value, especially in a small sample. We compared candidate models to obtain an optimal model based on model fitting criteria and clinical context. All analyses were conducted using SAS 9.3 (SAS Institute, Cary, NC).

#### Missing data

Patients who skipped an entire section of the YPAS were excluded. We performed imputations if patients answered at least one question in part I or at least one of the first seven questions in part II of the YPAS. Questions that were skipped in part I were assigned values of “0.” We imputed responses to skipped questions in part II of the YPAS by using subjects’ self-reported time spent participating in exertion-matched activities indicated on part I of the YPAS. Additionally, four patients did not specify the amount of daily time spent sitting as indicated in part II; since analogous data did not exist in part I of the YPAS, we assigned these missing values the cohort’s modal value for time spent sitting.

### Human studies

This study was approved by the IRBs at both the Brigham and Women’s Hospital and the Hospital General de la Plaza de la Salud. Written informed consent for participation in the study was obtained from patients.

## Results

### Recruitment

Of 170 subjects who received TJR from 2009–2012, 64 (38%) returned for follow-up, including 54.3% of subjects operated upon in 2012 and 2011, and 27.2% of patients operated upon in 2010 and 2009. No statistically significant differences were detected in age, sex, BMI, WOMAC pain and function scales, and SF-36 physical function and mental health scales between those who returned for a follow-up visit in 2013 and those who did not. The mean follow-up time for patients in this study was 2.1 years.

### Demographic and clinical characteristics of patients

The mean age of patients in the preoperative cohort who filled out the YPAS in 2013 was 61.1 years (SD = 13.89, range = 21 to 80 years). 81.3% of patients underwent TKR while the remainder received total hip replacement (THR). Two patients received both TKR and THR. The majority of patients were female (79.7%), unemployed (84.1%), and did not complete secondary school (80.6%). Approximately equal proportions of patients were married (35%) or single (31%). The majority lived with family members (90.3%). None of the patients in this analysis had experienced a serious complication of TJR surgery such as deep infection or deep vein thrombosis. Complete demographic and socioeconomic characteristics of the patients are shown in Table 1.

### Physical activity scores

For part I of the YPAS, the mean total time summary index (TTSI) for the 2009–2012 follow-up patients was 23.6 (SD: 29.1) hours per week. The mean energy expenditure summary index (EESI), which multiplies the subscales of the TTSI by varying intensity factors, was 5178.9 (SD: 6552.4) kcal per week. For part II of the YPAS, the mean ADSI was 45.2 (SD: 28.5), with a range from 7–123.

From components of the ADSI, times spent participating in moderate and vigorous intensity activities were derived for each patient. The mean time spent participating in vigorous PA was 84.6 (SD: 158.9) minutes per week, with a range of 0 to 603; the mean time spent ambulating was 103.2 (SD: 159.0) minutes per week, with a range of 0 to 610; the mean time participating in PA was 189.6 (SD: 233.1) minutes per week. 43.3% of follow-up patients met CDC/WHO criteria for adequate participation in aerobic PA.

### Bivariate analysis

#### Determinants of YPAS scores

The bivariate analysis did not reveal statistically significant predictors of the ADSI dimension of the YPAS score, though notable trends were observed (Table 2). Patients older than 65 at the time of surgery scored on average 11.5 points lower on the ADSI than patients younger than 65 (p = 0.130); patients who were married scored on average 10.9 points lower on the ADSI than patients who were single (p = 0.063); and patients who were unemployed scored on average 14.2 points lower on the ADSI than patients who were employed (p = 0.156).

**Table 2:** Preoperative characteristics associated in bivariate analyses with postoperative participation in PA 1–4 years post-TJR as measured by the ADSI of the YPAS

**Preoperative characteristics associated with physical activity level 1–4 years post-TJR**	**Mean ADSI score**	**(SD)**	**P value**	**Number of patients who meet CDC criteria**	**Number of patients who do not meet CDC criteria**	**Odds ratio**	**P value**
Social/socioeconomic characteristics							
Marital status			0.063				0.280
Single	56.9	31.6		11	7	2.9	
Married	46.0	24.9		8	12	1.2	
Divorced, separated, or widowed	35.1	27.2		7	13	REF	
Living situation			0.322				0.222
Alone	33.8	38.3		1	5	REF	
With family or friends	46.3	27.7		24	28	4.3	
Work status			0.156				0.737
Employed	57.1	34.3		5	5	REF	
Unemployed	42.9	27.2		21	28	0.8	
Education			0.793				0.397
Less than secondary school	42.3	22.3		12	11	2.9	
Secondary school, but did not graduate	48.0	35.6		10	14	1.9	
Graduated from secondary school or beyond	44.2	26.5		3	8	REF	
Psychological Factors							
Perception that surgery has a >90% chance of relieving pain and <1% chance of complication			0.080				0.151
Yes	49.8	28.5		20	19	2.7	
No	35.2	28.7		5	13	REF	
SF-36 mental health scale			0.405				1
≤68	40.2	20.7		7	9	1	
>68	47.3	31.2		19	24	REF	
Pain, Stiffness, and Functional Status							
WOMAC function score			0.792	12			0.603
<65	46.1	29.1				REF	
				
					19		
≥65	44.1	28.4		14	15	1.5	
WOMAC pain score			0.169			REF	0.795
<65	50.2	31.3			16		
				14			
≥65	39.9	24.8		12	18	0.8	
	44.1	33.3	0.791			REF	0.446
SF-36 physical activity score				11	18		
<15							
≥15	46.1	23.5		15	16	1.5	
Lower extremity joint burden score			0.236				1
≤1	50.3	29.8		11	14	REF	
≥2	41.4	27.3		15	20	1	
General medical characteristics							
Joint replaced			0.583				0.329
Hip	49.1	22.9		7	5	2.2	
Knee	43.9	30.2		18	28	REF	
Procedure			0.469				1
Unilateral	47.4	30.9		15	20	REF	
Bilateral	41.9	25.0		11	14	1	
Age			0.130				0.299
<65	51.3	28.3		15	14	REF	
≥65	39.8	28.5		11	19	0.5	
Year of surgery			0.224				0.096
2009	40.9	23.0		2	6	0.9	
2010	49.6	32.3		6	6	2.8	
2011	53.3	32.2		13	8	4.6	
2012	35.4	22.1		5	14	REF	
Sex			0.458				0.747
Female	43.7	28.7		20	28	0.7	
Male	50.7	28.2		6	6	REF	
BMI			0.327				0.018
<30	47.4	31.4		18	22	REF	
30-35	53.9	17.4		6	1	7.3	
≥35	27.0	7.50		0	4	N/A	
Smoking			0.404				1
Yes	28.5	13.4		1	1	REF	
No	45.9	29.0		25	32	0.8	
Consuming alcohol			0.29				0.167
Yes	36.1	23.7		2	7	REF	
No	47.5	30.0		23	24	3.4	
Self reported health status			0.434				0.219
Excellent or very good	38.9	26.2		5	11	0.3	
Good	43.8	27.6		7	12	0.4	
Fair or poor	50.9	30.4		13	9	REF	

#### Bivariate determinants of meeting CDC/WHO guidelines

**Figure 1:**
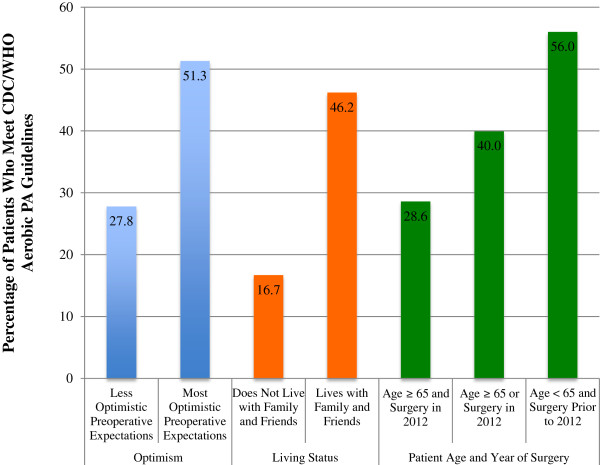
Percentage of patients who meet CDC/WHO aerobic PA guidelines stratified by selected patient characteristics.

Results of bivariate analysis suggest patients’ preoperative BMI was significantly associated with postoperative adherence to CDC/WHO guidelines for PA activity; 85.7% of patients with BMI of 30–35 met PA guidelines as compared with 45.0% of patients with BMI <30 and 0% of patients with BMI >35 (p = 0.018).Although no other variables reached statistical significance, three showed trends of clinically important effects. Patients living with family and friends were more likely to meet CDC/WHO guidelines for PA than those who live alone (OR 4.3; 95% CI 0.5-39.3). Patients who were the most optimistic in their preoperative expectations of both pain reduction and probability of complication were also more likely to meet CDC/WHO guidelines for PA (OR 2.7; 95% CI 0.8-9.2). Furthermore, patients who received surgery in 2012 were less likely to meet CDC/WHO guidelines than those patients who received the surgery in 2009–2011 (OR 0.3; 95% CI 0.1 to 1.1). Selected results of patients meeting CDC/WHO guidelines stratified by preoperative characteristics are displayed in Figure 1.

#### Bivariate determinants of ASDI

We used ANOVA to identify determinants of PA, with the calculated ADSI serving as the dependent variable. The initial variables analyzed were age, BMI, patient living status, preoperative expectations of surgery, alcohol consumption, year of surgery, joint operated on, and procedure (unilateral vs. bilateral). Four of these variables – year of surgery, age (dichotomized at 65), preoperative expectations of surgery, and living status – met the criteria for inclusion in the multivariate model. Age and year of surgery were moderately correlated (r = 0.36); to remove the intercorrelation, we created a variable that combined age (either greater than or less than 65) and year of surgery (either 2012 or prior to 2012).

#### Multivariate analysis

The model, which included 56 patients, demonstrated that patients who were both younger than 65 at the time of surgery and who received surgery prior to 2012 had adjusted mean ADSI 22.9 points higher than patients who were older than 65 and who had surgery in 2012 (adjusting for other variables in the model; p = 0.011; Table 3). Patients who lived with friends or family had adjusted mean ADSI 17.2 points higher than patients living alone (p = 0.135); patients who were the most optimistic preoperatively with regards to postoperative pain and postoperative complication had ASDI scores that were an adjusted mean of 19.8 points higher than those less optimistic (p = 0.011).

**Table 3:** Multivariate linear regression results including 56 patients

**Parameter**	**Estimate**	**Wald 95% confidence limits**	**P value**
**Intercept**	−18.8	−58.6, 21.1	0.356
**Age <65 and surgery prior to 2012**	22.9	5.3, 40.6	0.011
**Age ≥65 or surgery in 2012 (but not both)**	17.5	−1.2, 36.2	0.067
**Age ≥65 and surgery in 2012**	REF		
**Lives with family or friends**	17.2	−5.3, 39.7	0.135
**Lives alone**	REF		
**Optimistic preoperative expectations**	19.8	4.5, 35.0	0.011
**Not optimistic**	REF		

The estimate represents the number of ADSI points associated with the presence of the variable, adjusted for other variables in the model.

## Discussion

In this study we sought to measure participation in PA and identify preoperative predictors of PA among postoperative TJR patients in a developing country. Our results indicate that only 43.3% of Dominican patients 1–4 years post-TJR met the CDC/WHO guidelines for sufficient participation in aerobic PA. Additionally, while our small sample size precluded definitive identification of factors associated with PA, we identified several preoperative patient characteristics that demonstrated potentially clinically important effects on postoperative aerobic PA as measured by the ADSI of the YPAS. Specifically, patients were likely to have highest PA if they were younger preoperatively and had TJR performed more than two years prior; if they were most optimistic about the results of surgery in terms of pain relief and complications; and if they did not live alone. Furthermore, patients with BMIs ≥35 were significantly less likely to meet CDC/WHO guidelines for sufficient participation in aerobic PA.

To the best of our knowledge, this is the first study that has measured participation in aerobic PA in postoperative TJR patients in a developing country, and compared them against global recommendations. Recent data indicate that in an age-matched population in the United States, 50.9% of the population aged 55–64 and 52.7% of the population 65 and older met CDC criteria for participation in aerobic PA [25]; our Dominican cohort participates in less PA, with 43.3% meeting the CDC goal. However, it is important to note that these US patients are not postoperative TJR patients. TJR patients make substantial recovery progress, and in theory are provided a joint that enhances their functional capacity to perform PA. However, literature suggests that postoperative TJR patients may not reach pre-morbid levels of physical capacity [40,41]. One study demonstrated that physical deconditioning from habitual preoperative inactivity might contribute to decreased function in older adults [40]. Additionally our Dominican study population is culturally distinct from populations studied in the US. Others have noted cultural differences in participation in exercise as leisure, which may account for the comparatively lower level of participation in PA observed in this study [41]. In addition to cultural distinctions, there are likely differences in education, income, and general accessibility to recreational PA between the two populations, although these differences were not assessed in this study. Overall, it is important to note that patients generally achieved good pain relief and were satisfied [10]; thus, the modest improvements in PA should not be taken to mean the surgery did not help the Dominican patients.

Additionally, we identified several preoperative factors that had potentially clinically relevant associations with PA as measured by the ADSI of the YPAS. These findings fit well into the context of existing literature. It has been suggested that gradual improvements occur in TJR patients through at least two years postoperatively; this agrees with our finding that one-year postoperative patients were less physically active than those patients with more than one year of recovery [42]. Although patients who were operated upon before 2012 tended to participate in more PA, the 2009 cohort participated in less PA than the 2010 and 2011 groups. We are unsure of the reason for this, but we do note that the 2009 sample size is small. Furthermore, our finding does not vitiate the excellent functional outcomes of surgery documented in older patients. For example, it has also been documented that older patients are slower to regain functional capacity, although they do experience excellent long-term outcomes that are comparable to those enjoyed by younger TJR patients [43,44]. For older patients, surgery has been noted to improve the ability to participate in house cleaning and shopping [44]. Although our data suggest patients >65 are less likely to participate in PA, due to our small sample size, more research is required to make an assessment. Additionally, prior work has examined the links between recovery expectations and rehabilitation outcomes in patients with musculoskeletal disorders, suggesting that optimism is related to improved function and outcomes [45-47]. Furthermore, these patients may be more deconditioned preoperatively due to their long-standing arthritis. It is also important to note that a modestly higher level of PA was observed in the THR group; although this may simply be due to chance, this may also reflect the somewhat better functional and pain outcomes reported in THR patients vs. TKR patients [48].

These findings should be interpreted in the context of our study’s limitations. Our study is limited by small population size with only 64 participants. Although over 80% of Operation Walk Boston participants attend at least one annual follow-up visit, the attendance generally declines with each year of follow-up, accounting for the 38% return rate among all subjects who were eligible for follow-up in 2013. A larger sample would better tease out predictors of postoperative aerobic PA. Additionally, although the most precise way to measure PA is with accelerometers, this was not feasible in the developing world setting, so we used a validated questionnaire. Op Walk Boston patients had far advanced arthritis and substantial financial need; therefore, they may not be representative of the Dominican population suffering from arthritis. The proportion of patients attending the follow up clinics was limited, particularly those operated upon in earlier years, opening the possibility of selection bias. Furthermore, patients who underwent bilateral surgery were not asked about each joint individually. Finally, all of the operations were performed by American surgeons who operate on a high volume of patients; thus, the surgical outcomes may not be representative of the broader experience of TJR.

## Conclusions

The patients in the Dominican cohort participate in less PA than recommended by the CDC/WHO. Patients who are older than 65, recently postoperative, less optimistic about postoperative outcomes, have BMIs >35 and live alone seem to participate in less PA. Our findings suggest that TJR recipients in the developing world could be participating in greater amounts of moderate to vigorous aerobic PA; it is important to note that this can be achieved by brisk walking and would not require additional resources or physical therapy support. The data presented here should help inform discussions between providers and TJR patients about the health benefits and potential barriers of PA; we suggest that physicians prescribe exercise and PA routines to all TJR patients in the developing world. Additional research with larger samples should be performed to substantiate these findings.

## Abbreviations

OA: Osteoarthritis; TJR: Total joint replacement; PA: Physical activity; CDC: Centers for Disease Control; WHO: World Health Organization; WOMAC: Western Ontario and MacMaster Universities Arthritis Index; SF-36: 36 Question Short Form Health Survey; YPAS: Yale Physical Activity Survey; TTSI: Total time summary index; EESI: Energy expenditure summary index; ADSI: Activity dimensions summary index; THR: Total hip replacement; TKR: Total knee replacement.

## Competing interests

The authors declare competing interests. None of the authors has financial and/or personal relationships with other people or organizations that could potentially and inappropriately influence our work and conclusions.

## Authors’ contributions

SE was involved with the conception and design, collection and assembly of data, analysis and interpretation of the data, drafting of the article, and critical revision of the article for important intellectual content. YD was involved with the assembly of data, analysis and interpretation of the data, statistical expertise, drafting of the article, and critical revision of the article for important intellectual content. DS was involved with the conception and design, collection and assembly of data, and critical revision of the article for important intellectual content. RG was involved with the conception and design, administrative and logistical support, obtaining of funding, and critical revision of the article for important intellectual content. LA was involved with the conception and design, collection and assembly of data, obtaining of funding, and critical revision of the article for important intellectual content. JC was involved with the analysis and interpretation of data, provided her statistical expertise, and participated in critical revision of the article for important intellectual content. CB was involved with the collection and assembly of data and critical revision of the article for important intellectual content. TT was involved with the conception and design, administrative and logistical support, obtaining of funding, and critical revision of the article for important intellectual content. JK was instrumental in the conception and design, collection and assembly of data, logistical support, obtaining of funding, analysis and interpretation of the data, drafting of the article, and critical revision of the article for important intellectual content. All authors approved the final manuscript.

## Pre-publication history

The pre-publication history for this paper can be accessed here:

http://www.biomedcentral.com/1471-2474/15/207/prepub
